# Porcine β-defensin 2 inhibits proliferation of pseudorabies virus in vitro and in transgenic mice

**DOI:** 10.1186/s12985-020-1288-4

**Published:** 2020-02-03

**Authors:** Jing Huang, Yanhua Qi, Antian Wang, Chao Huang, Xiao Liu, Xi Yang, Lu Li, Rui Zhou

**Affiliations:** 1 State Key Laboratory of Agricultural Microbiology, College of Veterinary Medicine, Huazhong Agricultural University, Wuhan, 430070 China; 2 Key Laboratory of Preventive Veterinary Medicine in Hubei Province, Cooperative Innovation Center for Sustainable Pig Production, Wuhan, 430070 China; 3 Chongqing Academy of Animal Sciences, Chongqing, 402460 China; 4 Key Laboratory of Development of Veterinary Diagnostic Products, Ministry of Agriculture and Rural Affairs of the People’s Republic of China, Wuhan, 430070 China; 5 International Research Center for Animal Disease, Ministry of Science and Technology of the People’s Republic of China, Wuhan, 430070 China

**Keywords:** Porcine β-defensin 2, Pseudorabies virus, Antiviral ability, Transgenic mice

## Abstract

**Background:**

Porcine β-defensin 2 (PBD-2), produced by host cells, is an antimicrobial cysteine-rich cationic peptide with multi-functions. Previous studies have demonstrated that PBD-2 can kill various bacteria, regulate host immune responses and promote growth of piglets. However, the antiviral role of PBD-2 is rarely investigated. This study aimed to reveal the antiviral ability of PBD-2 against pseudorabies virus (PRV), the causative pathogen of Aujeszky’s disease, in PK-15 cells and in a PBD-2 expressing transgenic (TG) mouse model.

**Methods:**

In this study, the cytotoxicity of PBD-2 on PK-15 cells was measured by CCK-8 assay. PK-15 cells were incubated with PRV pre-treated with different concentrations of PBD-2 and PRV titers in cell culture supernatants were determined by real-time quantitative PCR (RT-qPCR). TG mice and wild-type (WT) mice were intraperitoneally injected with PRV and the survival rate was recorded for 10 days. Meanwhile, tissue lesions in brain, spleen and liver of infected mice were observed and the viral loads of PRV in brain, liver and lung were analyzed by RT-qPCR.

**Results:**

PBD-2 at a maximum concentration of 80 μg/mL displayed no significant cytotoxicity on PK-15 cells. A threshold concentration of PBD-2 at 40 μg/mL was required to inhibit PRV proliferation in PK-15 cells. The survival rate in PBD-2 TG mice was 50% higher than that of WT mice. In addition, TG mice showed alleviated tissue lesions in brain, spleen and liver compared with their WT littermates after PRV challenge, while viral loads of PRV in brain, liver and lung of TG mice were significantly lower than that of WT mice.

**Conclusions:**

PBD-2 could inhibit PRV proliferation in PK-15 cells and protect mice from PRV infection, which confirmed the antiviral ability of PBD-2 both in vitro and in vivo. The application of PBD-2 in developing anti-viral drugs or disease-resistant animals can be further investigated.

## Background

Defensins are a group of cationic microbicidal peptides, which are constituted of 18–45 amino acids with three intramolecular disulfide bonds formed between pairs of conserved cysteine residues [1]. Defensins in vertebrates are subdivided into α, β and θ defensins based on their spatial structure and disulfide connectivity [2, 3]. Within pigs, β defensin is the only subfamily of defensins which has been characterized so far [4–6]. Porcine β-defensin 2 (PBD-2) was first determined by sequence alignment with the existing sequence of porcine β-defensin 1 [6]. It was further identified that PBD-2 was distributed in different pig tissues using a polyclonal antibody against PBD-2 [7]. The antimicrobial activity of PBD-2 against gram-positive and gram-negative bacteria has been well described [8–10]. PBD-2 also exhibited immunomodulatory properties [11, 12]. Our recent research demonstrated that PBD-2 could alleviate inflammation through interacting with toll-like receptor 4 and suppressing the downstream NF-κB signaling pathway [13]. Additionally, it has been found that PBD-2 can attenuate proliferation of porcine reproductive and respiratory syndrome virus (PRRSV) in MA-104 cells [8]. Molecular docking evaluation has revealed that peptides derived from PBD-2 have the potential to be used to inhibit hepatitis E virus by interacting with its capsid protein [14]. However, the effect of PBD-2 on virus proliferation in vivo remains unknown.

Pseudorabies virus (PRV), an enveloped double-stranded DNA herpesvirus, is the etiological pathogen of Aujeszky’s disease causing meningoencephalitis and paravertebral ganglioneuritis in pigs [15]. The Bartha-k61 vaccine was brought to China from Hungary in the 1970s to prevent PRV infection, which has led to the successful control of PRV since 1980s [16]. However, PRV variants in Bartha-K61-vaccinated pigs have resulted in resurgence of PRV prevalence in China since late 2011 [17]. In China, with the infection rate being 13.14% in 2016, it still requires more efforts to achieve eradication of PRV [18]. In addition to vaccination development, studies on novel strategies to combat PRV are also of great significance.

On the basis of amino acid sequence alignment and molecular biological analyses, PRV resembles other alphaherpesviruses within the family *Alphaherpesvirinae* [15]. The antiviral abilities of defensins against alphaherpesviruses have been described previously. Some α-defensins with antiviral ability against herpes simplex virus (HSV) infection were characterized, such as human neutrophil peptide (HNP) 1–4, human α-defensin (HD) 5 and 6, rabbit α-defensin NP-1 and NP-2 [19–24]. In terms of β-defensins, human β-defensin (HBD) 3 and a synthetic β-defensin analog constituted by domains of HBD-1 and HBD-3 have been confirmed to inactivate HSV [20, 25, 26]. Bovine neutrophil β-defensin 3 has been found to show inhibitory activity against bovine herpes virus 1 [27, 28]. Besides, HBD-2 has been reported to inhibit varicella zoster virus infection [29]. Retrocyclins 1 and 2 and rhesus θ defensin 3 belonging to θ-defensins have been found to suppress HSV infection [30, 31]. Therefore, the utilization of defensins as drugs or target genes for generation of disease-resistant animals may be useful to control virus infection.

Although swine serves as the natural host and reservoir of PRV, mice are often utilized as a preferred model organism to study PRV infection because of their susceptibility to PRV. In this study, the effects of PBD-2 on the proliferation of PRV were investigated both in PK-15 cells and transgenic (TG) mice generated in our previous study [13]. The results confirmed the effectiveness of PBD-2 to suppress virus proliferation and provided novel ideas to control PRV infection.

## Materials and methods

### Cell line and virus

The PRV strain Ea, isolated from Hubei province of China, was used in this study [32]. Porcine PK-15 cells were maintained in Dulbecco’s Modified Eagle Medium (Thermo Fisher Scientific, Waltham, MA, USA) supplemented with 10% fetal bovine serum (FBS) (Thermo Fisher Scientific) in an atmosphere of 5% CO_2_ at 37 °C.

### Propagation and titration of PRV

PK-15 cells were used to propagate PRV. Briefly, 100 μL of virus stock was added to PK-15 cells which reached 70% confluence on a T75 cell culture flask for 1 h at 37 °C. After removal of virus inoculum, 15 mL of maintenance medium was added. When 80% cytopathic effect (CPE) was present, cells were harvested and repeatedly freeze-thawed before centrifugation. PRV was obtained in the supernatant and titration of PRV was measured and calculated using the Reed-Muench method in accordance with protocols described previously [33].

### Cytotoxicity assay

Cell Counting Kit-8 (CCK-8) (DOJINDO, Shanghai, China) assay was used to determine cell viability in accordance with the manufactures’ instruction. Briefly, 100 μL of PK-15 cell suspension (6000 cells/well) was inoculated in a 96-well plate. After 4 h in culture, cells were attached to the bottom of the plate. Different concentrations of PBD-2 ranging from 5 to 80 μg/mL were subsequently added into the plate without the presence of FBS. Cells were co-incubated with PBD-2 at 37 °C for 16 h before adding 10 μL of the CCK-8 solution. After another three-hour incubation, the absorbance was measured at 450 nm using a microplate reader (TECAN, Zürich, Switzerland). Cell viability (% of control) = [(absorbance of test sample - absorbance of blank)/(absorbance of control - absorbance of blank)] × 100%.

### Detection of antiviral effect of PBD2 in PK-15

Synthetic mature PBD-2 peptide (DHYICAKKGGTCNFSPCPLFNRIEGTCYSGKAKCCIR, ChinaPeptides, Shanghai, China) was used to evaluate its antiviral activity against PRV. PRV stocks diluted in DPBS were preincubated with PBD-2 of different concentrations (5 μg/mL, 10 μg/mL, 20 μg/mL, 40 μg/mL, 80 μg/mL) at 37 °C for 1 h. The mixture was then added to a 96-well plate with 10,000 cells in each well (MOI = 0.1), and the plate was incubated at 37 °C for another 1 h. The inoculums were discarded and wells were washed with PBS before adding fresh maintenance medium. After 48 h, cell culture supernatants were collected for viral DNA isolation using a Quick-DNA/RNA Viral Kit (Zymo Research, Irvine, CA, USA). The resulting viral DNA was subjected to real-time quantitative PCR (RT-qPCR) along with a standard plasmid. The plasmid containing a glycoprotein D (gD) gene, a highly conservative gene of PRV, was serially diluted and subjected to RT-qPCR to generate a standard curve using the primers P1 (5′-CATCCTCACCGACTTCAT-3′) and P2 (5′-TACCAGTAGTTCACCACC-3′) for amplifying a part of gD gene which is 192 bp in length. The number of PRV copies was determined from the standard curve by converting the corresponding Ct value.

### Mice grouping and viability test of PRV infection

C57/BL TG mice expressing PBD-2 were generated by our lab previously [13]. Genomic DNA from mouse tail samples was extracted and used for identification of TG mice expressing PBD-2 by using primers NP03 (5′-GCTGGTTGTTGTGCTGTCTC-3′) and NP04 (5′-AGGTCCCTTCAATCCTGTTG-3′). At 6–8 weeks of age, TG mice (*n* = 4) weighing 20 ± 2 g were intraperitoneally injected with 0.2 mL of PRV (2 × 10^3.3^ TCID_50_/mL) each. Their wild-type (WT) littermates (*n* = 4) were treated with the same amount of PRV each. Survival was assessed in two groups of mice daily for 10 days.

### Quantification of PRV copies in mice tissues

Both TG and WT mice intraperitoneally injected with 0.2 mL of PRV (2 × 10^3.3^ TCID_50_/mL) were sacrificed on day 5 post-infection and brain, spleen, liver and lung were harvested for further analyses. DNA from 30 mg of brain, liver and lung tissues each was extracted using E.Z.N.A Tissue DNA kit (Omega Bio-tek, Norcross, GA, USA) according to the manufacturer’s instructions. The PRV copies in the total DNA of tissue samples were measured by RT-qPCR and calculated as described above.

### Histopathological analysis

Brain, spleen and liver tissues from TG and WT mice were fixed in PBS-buffered 4% formaldehyde for 48 h at 37 °C. Tissues were than embedded in paraffin and sliced into sections of 5 μm thickness. After that, sections were subjected to haematoxylin and eosin (HE) staining. Tissue lesions were observed under a microscope.

### Statistics

Statistical analyses were performed with GraphPad Prism 5 (GraphPad Software, La Jolla, CA, USA) using unpaired one-tailed Student’s *t*-test and shown as mean ± SD. *P < .05, **P < .01, ***P < .001, ****P < .0001.

## Results

### Antiviral effect of PBD-2 in PK-15 cells

**Fig. 1:**
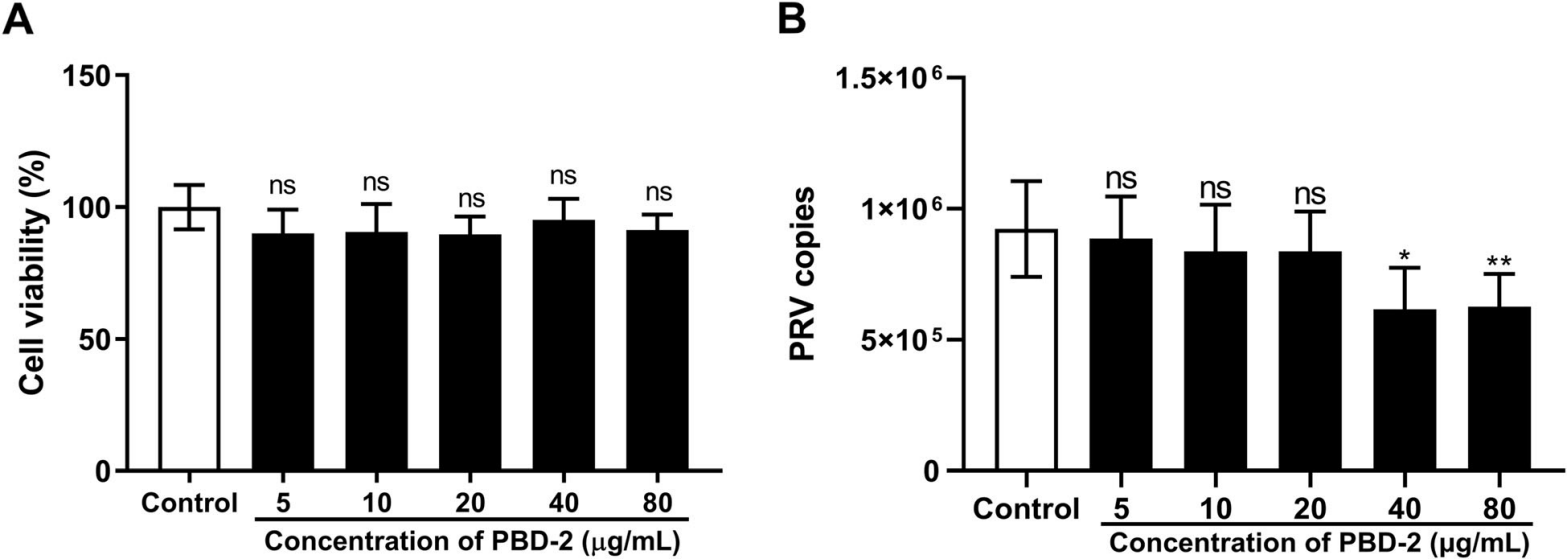
Effect of porcine β-defensin 2 (PBD-2) on PK-15 cells. **a** Cytotoxicity of PBD-2 on PK-15 cells. PK-15 cells were incubated with different concentrations of PBD-2 for 16 h (5, 10, 20, 40, 80 μg/mL). The cytotoxicity was measured by CCK-8 assay (*n* = 8); **b** Pseudorabies virus (PRV) was pre-incubated with PBD-2 of different concentrations and used to inoculate cells, the PRV copies were measured 2 days after infection (*n* = 5). Data are presented as mean ± SD from three independent experiments. * *P*<0.05, **P<0.01, ns = no significance, unpaired one tailed Student′s *t*-test

The cytotoxicity of PBD-2 on PK-15 was evaluated using CCK-8 assay. The cell viability analysis indicated that PBD-2 displayed no significant cytotoxicity on PK-15 cells at a maximum concentration of 80 μg/mL and at a minimum concentration of 5 μg/mL (Fig. 1a). Then, PBD-2 of different concentrations were used to assess the antiviral effect against PRV in PK-15 cells. Compared with PRV without pretreatment of PBD-2, significant reduction in viral titer was observed when PRV was pre-incubated with 40 μg/mL and 80 μg/mL of PBD-2 (*P* < .05) (Fig. 1b). These results indicated that PBD-2 at a relatively high concentration could significantly inhibit PRV proliferation in PK-15 cells without cytotoxicity activity.

### Survival rate analysis of TG mice after infection of PRV

**Fig. 2:**
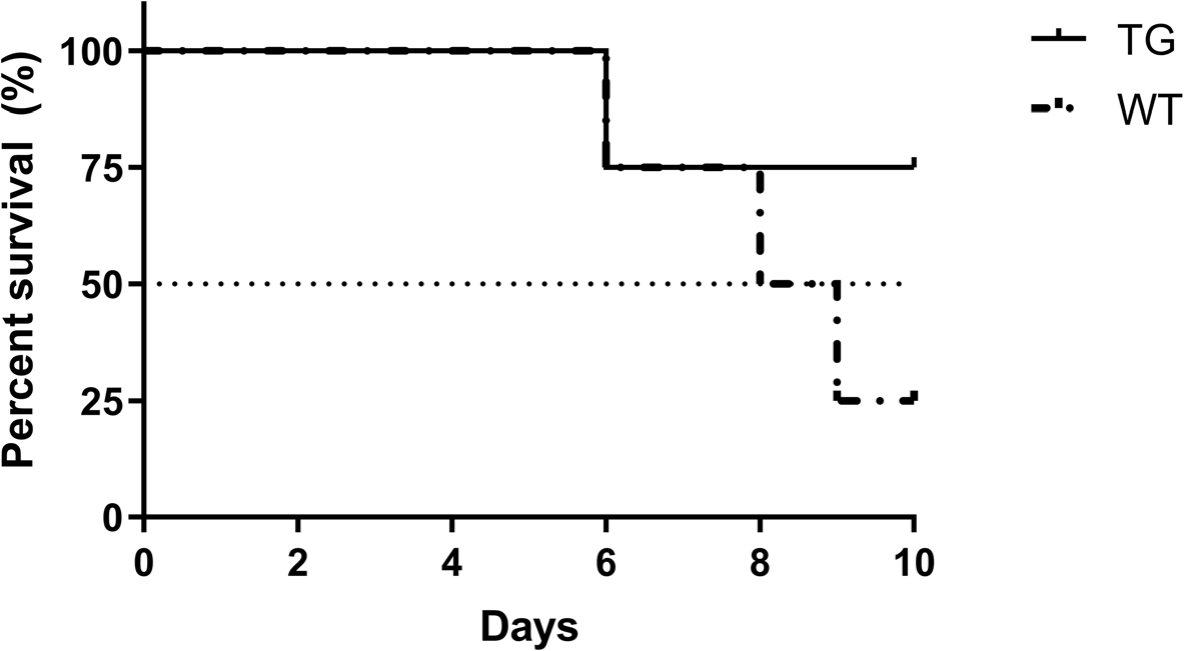
Survival of transgenic mice (TG) expressing PBD-2 and wild-type (WT) mice after PRV infection (*n* = 4 for each group). The mice were intraperitoneally injected of 0.2 mL of PRV (2 × 10^3.3^ TCID_50_/mL). This result represents three independent experiments with similar results

The antiviral effect of PBD-2 on the survival of mice infected with PRV was investigated. WT mice intraperitoneally infected with 0.2 mL of PRV (2 × 10^3.3^ TCID_50_/mL) showed a survival rate of 25% while expression of PBD-2 increased the survival rate of TG mice to 75% (Fig. 2). Thus, the results revealed that TG mice expression PBD-2 were more resistant to PRV infection than control mice.

### Viral loads in TG mice after PRV infection

**Fig. 3:**
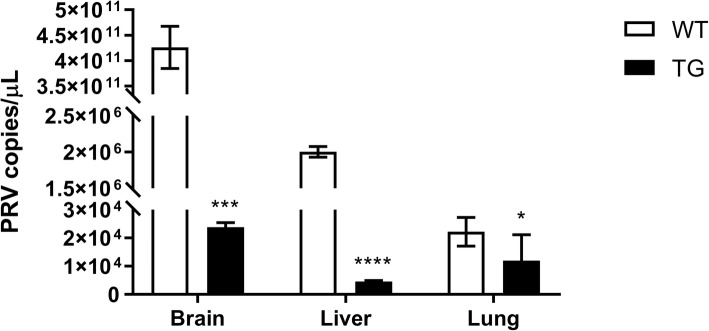
Viral loads of PRV in tissue samples of mice infected with PRV. Total DNA used to determine PRV copy numbers were obtained from brain, liver and lung tissues of WT and TG mice (*n* = 4 for each group) on day 5 after PRV injection. Data are presented as mean ± SD and are plotted from three independent experiments. *P<0.05, ***P<0.001, ****P<0.0001, unpaired one tailed Student′ s *t*-test with Welch′ s correction

The PRV genomic copy numbers were quantified in brain, liver and lung tissues of TG and WT mice infected with PRV by RT-qPCR. PRV viral loads in brain tissues of TG mice and WT mice were higher than those in liver and lung tissues. However, PRV viral loads in brain (*P* < .001), liver (*P* < .0001) and lung (*P* < .05) of TG mice were significantly lower than that of WT mice (Fig. 3). Hence, PBD-2 could significantly depress viral loads of PRV in different tissues.

### Histopathological analysis of TG mice after infection of PRV

**Fig. 4:**
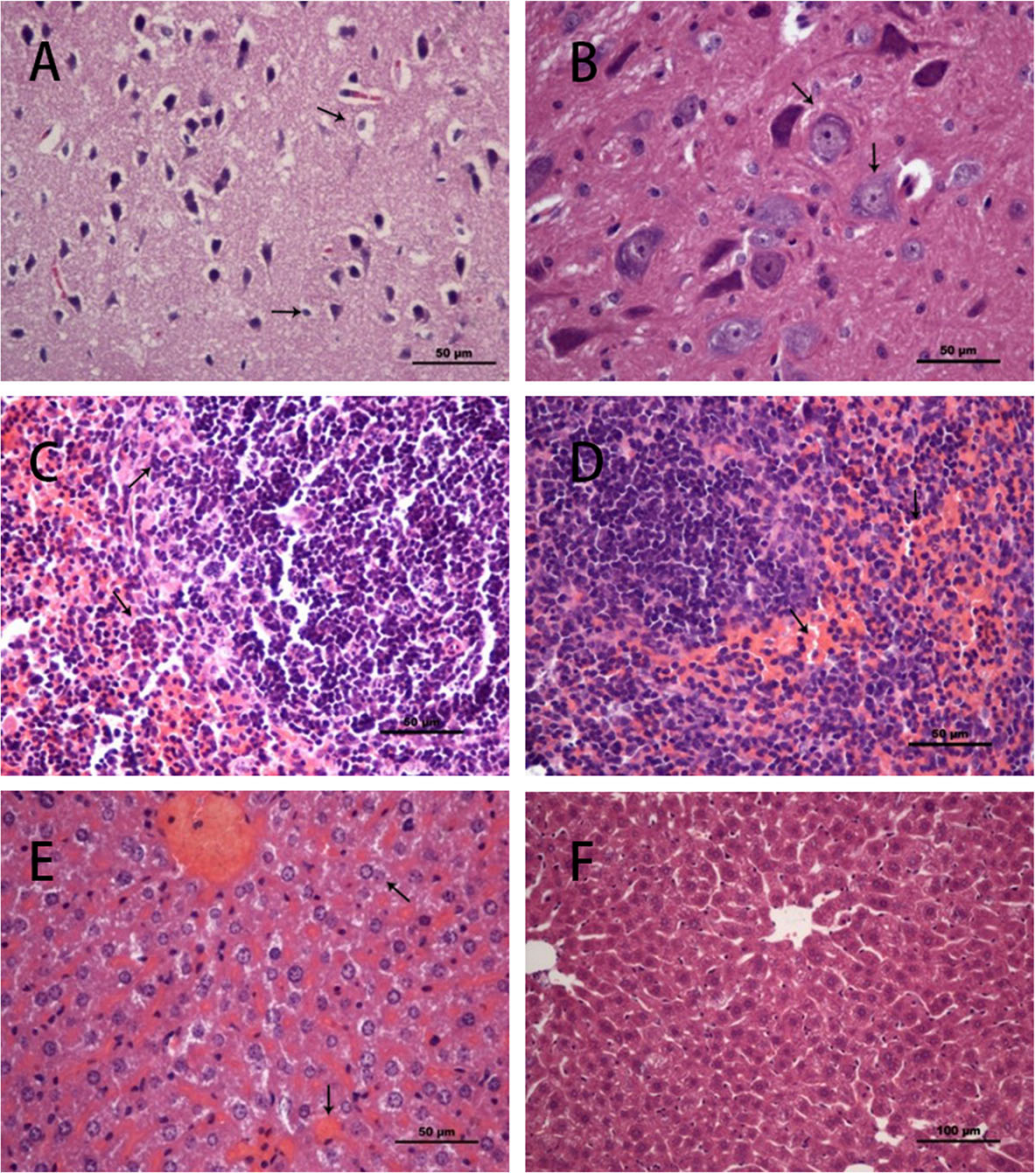
Histopathological analysis on brain, spleen and liver tissues of TG and WT mice infected with PRV. Tissues were fixed in 4% formaldehyde and subsequently embedded paraffin for sectioning and HE staining. Black arrows indicate histopathological damages. **a**, **c** and **e** showed HE stained brain, spleen and liver tissues of represented WT mice; **b**, **d**, **f** showed HE stained brain, spleen and liver tissues of represented TG mice

The pathological changes of WT mice and TG mice after PRV infection were also detected. Microglia nodules associated with neuronal degeneration and necrosis were observed in brain tissue of WT mice (Fig. 4a) while neuron swelling, dissolution of Nissl bodies and basophilic inclusions were present in TG mice (Fig. 4b). Besides, necrosis of cells was found in spleen tissue of WT mice (Fig. 4c) and TG mice showed a slight hyperemia in spleen (Fig. 4d). Degeneration of cells and congestion in the liver of WT mice were observed (Fig. 4e) while there were no apparent lesions in that of TG mice (Fig. 4f). Together, these results demonstrated that TG mice displayed milder tissue lesions than WT mice after PRV infection.

## Discussion

The impact of PBD-2 on viral pathogenicity has not been well examined experimentally yet. A previous study has showed that PBD-2 exhibits antiviral ability against PRRSV when the concentration reaches 64 μg/mL [8]. Similarly, in this study, PBD-2 at 40 μg/mL and 80 μg/mL was found to inhibit PRV proliferation in PK-15 cells while no significant effect was observed for lower concentrations of PBD-2 (Fig. 1b). These indicate that a threshold concentration is required for PBD-2 to inhibit viral proliferation. According to the survivability, detectable virus copies and pathological changes, the TG mice displayed significantly increased resistance to PRV infection compared with the WT mice. Therefore, the antiviral effect of PBD-2 was confirmed both on cells and in TG mice expressing PBD-2. It has been revealed that defensins can direct inactive enveloped viruses by disrupting viral lipid bilayers [34]. In this study, PRV was preincubated with PBD-2 and then used to infect cells. According to the result, we hypothesize that PBD-2 might have a direct virucidal effect on PRV by damaging the viral envelopes. Besides, it may also affect PRV entry because PBD-2 was still present during the whole process of viral adsorption. Since PRV shared high structural and sequence similarity with other alphaherpesviruses [15], the antiviral mechanisms of other defensins against alphaherpesviruses would help understand how PBD-2 inhibited PRV proliferation. HNP-4 and HD-6 were capable of block HSV binding by interacting with heparan sulfate, the primary receptor for HSV binding, while HBD-3 with enhanced inhibitory effect against HSV bound both heparan sulfate and gB [20]. HD-5 was able to prevent viral adhesion and entry of HSV by binding to both gB and gD [20, 21, 23]. Likewise, rabbit NP-1 and HNP-1, -2, -3 inactivated HSV by preventing viral entry [22, 31, 35]. Retrocyclin 2 prevented HSV binding, entry and intercellular spread by binding carbohydrate moieties on glycoprotein B [31]. In general, these defensins prevent HSV infection by blocking viral binding, penetration or both. Whether PBD-2 is able to block viral binding and penetration by interacting with specific host membrane receptor(s) can be further studied.

Defensins have been confirmed to be effective to resist pathogens. Chen et al. argued that higher expression levels of PBD-2 and other β-defensins in Meishan pigs than those in crossbred pigs conferred stronger immunity of Meishan pigs against diseases [36]. Our previous study revealed that PBD-2 overexpressing pigs and PBD-2 TG mice showed enhanced resistance to bacterial infection [10, 13]. In this study, we further verified that PBD-2 TG mice displayed increased resistance to viral infection. Thus, PBD-2 can be a useful target to generate disease-resistant animals against both bacterial and viral infections. PRV is an important pig pathogen which is still present in China, Japan, USA and some European countries [37, 38]. The inhibitory effect of PBD-2 in vitro and in vivo discovered in this study provided possible new strategies to control PRV, such as overexpressing PBD-2 in pig tissues. In transgenic mice infected with PRV, PBD-2 could reduce viral loads and alleviated tissue lesions at different degrees in different tissues (Figs. 3 and 4). Our previous study has discovered that PBD-2 expression level in organs differed in TG mice [39], which suggests a correlation between PBD-2 expression level and its anti-PRV effect in different organs. It is worth noting that PRV loads of brain tissues (Fig. 3) were the highest and damages of brain tissues were the severest (Fig. 4) either in TG mice or in WT mice, confirming that PRV had a distinct tissue tropism [15]. Herein, increasing PBD-2 expression in animals, especially in brains, using gene-editing techniques might promote the resistance of animals to PRV in vivo.

On the other hand, PBD-2 may be used as drug additives or vaccine adjuvants to develop alternative strategies to control PRV and other viruses. Attempts at using β-defensins to control viral diseases in vivo have been carried out. Expression of zebrafish β-defensin 2 in fishes improved their resistance to spring viraemia of carp virus infection [40]. Application of murine β-defensin 2 as a DNA vaccine adjuvant protected mice against H5N1 avian influenza viruses [41]. In addition, mice intranasally or intratracheally inoculated with P9, a derivative of mouse β-defensin 4, were protected from infections of three types of influenza A viruses and SARS-associated coronavirus [42]. Besides, intraperitoneal injection of recombinant mouse β-defensin 3 (rMBD3) in mice was found to alleviate coxsackievirus B3-induced myocarditis [43]. Similarly, intravenous injection of rMBD3 improved the survival rate of mice infected with influenza A virus [44].

## Conclusions

In summary, within this study, the antiviral effect of PBD-2 in vitro and in vivo were confirmed using PRV infection model. The technique of large-scale production of PBD-2 [45] and the existing transgenic pigs overexpressing PBD-2 [10] have created possibilities for the application of PBD-2 as novel prophylactic and therapeutic methods against Aujeszky’s disease and other viral diseases. In addition, it is worthy of further studies on the underlying mechanism for the inhibitory effect of PBD-2 on PRV, which will offer more insights regarding antiviral effects of defensins.

## Data Availability

Detailed data will be available on request.

## Abbreviations

CCK-8: Cell counting kit-8
CPE: Cytopathic effect
FBS: Fetal bovine serum
gD: Glycoprotein D
HBD: Human β-defensin
HD: Human α-defensin
HE: Haematoxylin and eosin
HNP: Human neutrophil peptide
HSV: Herpes simplex virus
PBD-2: Porcine β-defensin 2
PRV: Pseudorabies virus
rMBD-3: Recombinant murine β-defensin 3
TG: Transgenic
WT: Wild-type
